# Analysis of differentially expressed genes and adaptive mechanisms of *Prunus triloba* Lindl. under alkaline stress

**DOI:** 10.1186/s41065-017-0031-7

**Published:** 2017-05-04

**Authors:** Jia Liu, Yongqing Wang, Qingtian Li

**Affiliations:** 1 College of Horticulture, Sichuan Agricultural University, Chengdu, Sichuan 611130 People’s Republic of China; 2 Horticulture Research Institute, Sichuan Academy of Agricultural Sciences, Chengdu, Sichuan 610066 People’s Republic of China; 3 Southwestern Key laboratory of Horticultural Crops Biology and Germplasm Enhancement, Ministry of Agriculture, Chengdu, Sichuan 610066 People’s Republic of China; 4 State Key Lab of Plant Genomics, Institute of Genetics and Developmental Biology, Chinese Academy of Sciences, Beijing, 100101 People’s Republic of China

**Keywords:** Alkaline stress, Differentially expressed genes, *Prunus triloba* Lindl, Physiological analysis, RNA sequencing

## Abstract

**Background:**

*Prunus triloba* Lindl. is a naturally salt-alkaline-tolerant plant with several unique characteristics, and it can be used as the rootstock of Chinese plum (*Prunus salicina* Lindl.) in saline-alkaline soils. To comprehensively investigate the alkaline acclimation mechanisms in *P. triloba*, a series of analyses were conducted under alkaline stress, including analyses of the kinetics of molecular and physiological changes, and leaf microstructure.

**Results:**

To understand the kinetics of molecular changes under short-term alkaline stress, we used Illumina HiSeq 2500 platform to identify alkaline stress-related differentially expressed genes (DEGs) in *P. triloba*. Approximately 53.0 million high-quality clean reads were generated from 59.6 million raw reads, and a total of 124,786 unigenes were obtained after *de novo* assembly of *P. triloba* transcriptome data. After alkaline stress treatment, a total of 8948 unigenes were identified as DEGs. Based on these DEGs, a Gene Ontology (GO) enrichment analysis was conducted, suggesting that 28 genes may play an important role in the early alkaline stress response. In addition, analysis of DEGs with the Kyoto Encyclopedia of Genes and Genomes (KEGG) revealed that pathways were significant at different treatment time points. A significant positive correlation was found between the quantitative real-time PCR (qRT-PCR) results and the RNA-Seq data for seven alkaline-related genes, confirming the reliability of the RNA-Seq results. Based on physiological analysis of *P. triloba* in response to long-term alkaline stress, we found that the internal microstructures of the leaves of *P. triloba* changed to adapt to long-term alkaline stress. Various physiological indexes indicated that the degree of membrane injury increased with increasing duration of alkaline stress, affecting photosynthesis in *P. triloba* seedlings.

**Conclusions:**

This represents the first investigation into the physiology and transcriptome of *P. triloba* in response to alkaline stress. The results of this study can enrich the genomic resources available for *P. triloba*, as well as deepening our understanding of molecular and physiological alkaline tolerance mechanisms in *P. triloba*. This will also provide new insights into our understanding of alkaline acclimation mechanisms in Chinese plum (*Prunus salicina*) trees.

**Electronic supplementary material:**

The online version of this article (doi:10.1186/s41065-017-0031-7) contains supplementary material, which is available to authorized users.

## Background

Chinese plum (*Prunus salicina* Lindl.) is a small deciduous tree that belongs to the genus *Prunus* in the Rosaceae family. The wild peach (*Prunus persica* Lindl.) is widely used as its rootstock in the area south of the Yangtze River in the provinces of Yunnan, Guizhou, and Sichuan; in the arid area of the northwest district; and in Henan, Hebei, Shandong, and other areas in China. While the wild peach possesses the characteristics of high adaptability, developed roots, fast growth, good grafting affinity, and rapid germination speed [1], it also possesses some less positive characteristics. The major drawback of the wild peach is its poor saline-alkaline resistance. When the soil pH reaches 7.5–9.0, iron chlorosis generally occurs in plant tissues. This not only causes plants to oxidize available Fe^2+^, leading to the precipitation Fe(OH)_3_, but also influences other iron dissolution pathways and reduces the stability of chelated iron, leading to iron malabsorption [2, 3].

The central hill area in the Sichuan Basin is the main planting region for plum trees in China, and it widely consists of calcareous purple soils. The pH values of these soils range from 7.69 to 8.47, and iron chlorosis is common among fruit trees in this area. Iron chlorosis occurs easily in regions with alkaline soil; when fruit trees are under alkaline stress for an extended period of time, they grow poorly, fail to grow flowers or fruits, and may even die, having a detrimental effect on the fruit tree industry [2]. Recently, several methods have been explored for improving saline-alkaline soils [4, 5]. Among these, the selection of an appropriate saline-alkaline-tolerant plant suited to the type of saline-alkaline soil is the main method used to cultivate plants on saline-alkaline land [5]. Therefore, it is important to study the effect of alkaline damage of various plants and to select alkaline-resistant varieties of fruit trees or improve the alkaline resistance of fruit trees. However, the alkaline tolerance of fruit trees is actually dependent on the rootstock alkaline tolerance. Because the rootstock variety directly affects the fruiting time, yield, and lifetime of fruit trees, so the screening of alkaline-tolerant rootstocks is an effective way to improve the alkaline resistance of fruit trees.

*Prunus triloba* Lindl., also known as flowering plum or flowering almond, is a deciduous and flowering shrub or small tree species of the genus *Prunus* (family Rosaceae) native to northeastern, northwestern, and northern China [6, 7]. *P. triloba* is a popular ornamental plant in China, especially known as an important early spring flowering ornamental in the landscape of northern China. There are many varieties of *P. triloba*, and it has been cultivated for more than 300 years [6, 8]. It is an octaploid with a karyotype of 2n = 8x = 64, and it possesses the characteristics of good adaptability, tolerance to poorly drained soils and to low level management, tolerance to cold stress and to drought stress, and high disease resistance [6, 9, 10]. In addition, *P. triloba* is a naturally salt-alkaline-tolerant plant, and it grows well in saline-alkaline soils with a pH of 8.8 (0.3% salt content). *P. triloba* has good grafting affinity with Chinese plum, bears fruit early, results in high yield, suggesting that it can be used as the rootstock of *P. salicina* in the middle hill region in the Sichuan Basin [9].

A range of abiotic and biotic stresses can severely restrict plant growth and reduce crop productivity, of which soil salinity, alkalinity, or saline-alkalinity are the most common abiotic stresses encountered by plants [11]. These types of soils are widely distributed across more than 100 countries [12]. The salinization of soil is usually caused by neutral salt (NaCl, Na_2_SO_4_, etc.), while the alkalinization of soil is usually caused by alkaline salt (NaHCO_3_, Na_2_CO_3_, etc.) and soil saline-alkalinity is caused by the presence of both neutral and alkaline salts [13]. In contrast to saline soil, which mainly affects plants through osmotic stress and ion toxicity, alkaline soil damages plants through high pH [14]. Previous research showed that the negative effect of soil alkalinization on plants was greater than that of soil salinization [11, 13]. While there have been a number of reports on the mechanisms of plant tolerance to salinity [15], much less attention has been paid to the plant response to alkaline stress. Therefore, analysis of the underlying mechanisms of soil alkalinity on plant growth and development are urgently needed, especially for commercial crops.

Plant stress resistance is not the result of a single mechanism of action but rather is a complex stress response borne of various aspects of cell, physiological, and molecular biology. The physiological and biochemical mechanisms of plant abiotic stress tolerance have widely been studied by researchers, and the underlying molecular mechanisms have always been the focus of plant biology research area. The molecular mechanisms of plant abiotic stress tolerance involve multiple genes and complex genetic regulatory networks [16]. Only by understanding the functions of these stress-inducible genes can we decipher the related physiological, biochemical, and molecular mechanisms of stress tolerance [16]. Of late, emerging high-throughput techniques have become a significant means of achieving this goal. RNA sequencing (RNA-Seq) technology is frequently used to study plant stress tolerance because it is an economical, sensitive, and efficient tool for the identification and analysis of key stress-responsive genes. In addition, it can be used for non-model species [17–19].

Therefore, in order to comprehensively investigate alkaline acclimation mechanisms in *P. triloba*, a series of analyses were performed under alkaline stress, including assessment of the kinetics of molecular and physiological changes, and leaf microstructure. The Illumina HiSeq 2500 platform was used to examine the transcriptomes of leaf samples from alkaline-treated or control *P. triloba* seedlings. We then *de novo* assembled a transcriptome library of *P. triloba* to identify differentially expressed genes (DEGs) from plants under alkaline stress. To our knowledge, this is the first physiological and transcriptomic analysis of *P. triloba* in response to alkaline stress. Our results will enrich genomic resources for *P. triloba* and deepen our understanding of molecular and physiological alkaline tolerance mechanisms in *P. triloba*. Our results also will provide new insight into understanding alkaline acclimation mechanisms in Chinese plum (*P. salicina*) trees.

## Methods

### Plant materials and alkaline stress treatment conditions

The seeds of *P. triloba* were preserved in low temperature sand in November 2014. On the middle ten days of the second month of the second year, the seeds of *P. triloba* were sown in the earthenware pots, about 25 cm in diameter, and with holes in the bottom. Each pot was filled with equal amount of soils, which were fully mixed by peat soil and turfy soil in the ratio of 1 to 1. When the height of seedlings reached to about 15 cm, 120 plantlets with similar size and growth, housing outdoor with a transparent plastic film at the top to keep out the rain, were selected to subject to alkaline stress treatments as the test materials, and other 30 plantlets were selected as control group. The plantlets of alkaline stress treatments were watered using alkaline liquor, which was established by mixing NaHCO_3_ and Na_2_CO_3_ at 9:1 molar ratio, and the concentration and pH value of the solution were 50 mmol/L and 9.11 ± 0.104, respectively. The alkaline solution was firstly used to water the test materials at 16:00 sunny day. Thereafter, the alkaline solution was poured every 3 days, and every time until the pH of effluent from the bottom of pot reached more than 9.0. The control group was simultaneously with the same amount of water irrigation. The alkaline treatment lasted for 12 days.

### *De novo* transcriptome analysis of *P. triloba* in response to short-term alkaline stress

#### RNA isolation, cDNA library construction and sequencing

We separately sampled the leaf blades at 0, 1, 3, 6, and 12 h after the alkaline stress. The harvested leaf samples were immediately frozen in liquid nitrogen for RNA extraction. This process was performed in two biological replicates at the each time point, of which each replicate consisted of 10 pooled leaves. The achieved ten *P. triloba* samples were isolated using the Universal Plant RNA Extraction Kit (Aidlab, Beijing, China) in accordance with the manufacturer’s protocol. RNA purity, concentration, and quality were examined with a Nanodrop 2000 UV-Vis spectrophotometer (Thermo Fisher Scientific Inc., USA), Qubit® 2.0 Fluorometer (Life Technologies, USA) and Agilent 2100 Eukaryote Total RNA Nano Kit (Agilent, Santa Clara, CA, USA), respectively.

Total 10 RNA samples of *P. triloba* were then delivered to GENEWIZ, Inc. (Suzhou, China) for cDNA library construction and sequencing. In briefly, cDNA library was built for each of 10 RNA samples using the NEBNext Ultra RNA Library Prep Kit for Illumina (New England Biolabs, Ipswich, MA, USA). In order to ensure the quality of the library, a Beckman AMPure XP Bead Kit (Beckman Coulter, Danvers, MA, USA) and Agilent 2100 High Sensitivity DNA Kit (Agilent) were then used to purify and to detect the quality of the library, respectively, and the effective concentration of the library was accurately quantified with a ABI 7500 real time PCR system. Subsequently, cBOT automatic clustering of these qualified cDNA libraries were conducted using TruSeq PE Cluster Kit V3 (Illumina). Finally, High-throughput sequencing was sequenced on Illumina HiSeq 2500 platform by synthesis.

#### De novo transcriptome assembly

The obtained sequencing raw data in FASTq format were initially processed by Trimmomatic (v0.30) [20] to get high-quality clean data, which trimmed reads containing adaptors, filtered off low quality reads (bases with quality scores<20 at the 3′ or 5′ end) and reads with poly-N, and removed sequences shorter than 20 bp. The clean reads from each library were evaluated with the Q20 (the percentage of bases with a Phred value >20), Q30 (the percentage of bases with a Phred value >30), GC-content, and N-content, and were checked using fastqc (http://www.bioinformatics.babraham.ac.uk/projects/fastqc/). Then, *de novo* assembly of the clean reads was achieved using the Trinity assembler (version r2013-02-25) [21]. The software TGICL [22] was further used to splice sequences and redundancy analysis in order to produce longer, more complete non-redundant sequences which were called unigenes. The software TransDecoder [23] was introduced to predict the open reading frames (ORFs) of transcripts and to extract longest open-ended ORFs. Finally, the clean reads were mapped to the unigenes using Bowtie2 (v2.1.0) [24] with the default parameters.

#### Quantification of gene expression levels and differential expression analysis

The gene expression level of the assembled unigenes of each sample was separately estimated by software RSEM (V 1.2.4) [25] using the FPKM (fragments per kilobase per million reads) method. Differential gene expression analysis of all samples after different periods of alkaline stress was performed using the R package DESeq (V 1.14.0) based on the negative binomial distribution [26] to identify differentially expressed genes (DEGs) in *P. triloba* treated with alkaline stress. The *P*-value corresponded to the differential gene expression test. A False discovery rate (FDR) method was used to determine the threshold of the *P*-value. Genes with a FDR threshold ≤ 0.05 and |log2 (fold change)| > 1 found by DESeq were designated as differentially expressed. Afterwards, GO functional categories were assigned to differentially expressed genes implementing by the R package GOseq based on the Walleius non-central hyper-geometric distribution [27]. DEGs also underwent KEGG pathway enrichment analysis in order to systematically understand the biological pathways involved in the DEGs using software KOBAS [28].

#### Gene validation of qRT-PCR analysis

To validate the DEGs detected by RNA-Seq, seven unigenes, which were determined to be significantly related to alkaline stress, were subjected to real-time quantitative reverse transcription-PCR (qRT-PCR) analysis. The RNA samples were extracted from the leaf tissues of *P. triloba* materials at 0 h, 3 h, 6 h, and 12 h after the alkaline stress using the same method described above. A TUREscript 1st Stand cDNA SYNTHESIS Kit (Aidlab Biotechnologies Co., Ltd, Beijing, China) was used to synthesize the cDNAs as described by the manufacturer using an Oligo (dT) Primer with 500 ng total RNA. Specific primers designed for the seven selected genes were listed (Table 1). Real-time RT-PCR of the seven genes in technical and biological triplicates was performed using 2×SYBR® Green Supermix (DBI® Bioscience, Germany) in an optical 96-well plate with a qTOWER2.2 Real-Time PCR System (Anlytik Jena AG, Germany) in final reaction volumes of 25 μL, each containing 5 μL 1× 2×SYBR® Green Supermix (DBI® Bioscience, Germany), 0.5 μL 200 nM of the reverse primer and sense primer, 1 μL cDNA template, and 3 μL ddH_2_O. The thermal cycling conditions were as follows: 95 °C 3 min; 39 cycles of 95 °C for 10 s, 60 °C for 30 s, and then increasing the temperature to 95 °C (+1 °C/cycle, holding time 4 s). The internal reference gene was the actin gene. The relative expression level of each gene was determined using threshold cycles, by the 2^-△△^Ct method.

**Table 1:** Primer information for qRT-PCR

Gene Ontology	ID	Upstream / downstream primer (5′-3′)
AP-2 complex subunit alpha	c78498_g1_i1	CACTGGTATAGGCTGAAGAA
		TTCGGTAACAATATGGTCAATG
ALDH18A1, P5CS	c80260_g1_i1	TGGTATCTTAGTGGACTC
		AACTTATTGGACTTGTGAA
mitogen-activated protein kinase 1/3	c81662_g1_i1	GTGTTGCTGTTGGTGTTG
		CTTCTTCCTCCTCCTCTCA
aroDE, DHQ-SDH	c84090_g1_i1	GTAGTGTCAGTAGGTATGGAGAG
		GAGGTTAGGTGCGTGGAT
AP2-like factor, ANT lineage	c85502_g2_i1	GCAGCATCATAAGGAATC
		GGCAGCAACAATATCAAT
trehalose 6-phosphate synthase/phosphatase	c85846_g1_i3	GCCAGCATATTGTTGAAG
		CCATCATTGACCATTCTTG
mitogen-activated protein kinase kinase 1	c88652_g1_i2	CATCTATCTGTGGAAGGA
		GCATCTGAATAGGAATCTG
	ACTIN	GCCGAGTATGATGAATCTG
		GAGTCTGCCTATCCTTGA

### Physiological analysis of *P. triloba* in response to long-term alkaline stress

#### Effects of long-term alkaline stress on the leaf microstructure of *P. triloba*

The upper leaf blades of the test materials on 0 d and 12 d after the alkaline stress were sampled to immediately use for observing the blade structure through the microscope. The center area (5 mm × 5 mm) of both sides of leaves avoided the leaf vein were sampled by one side blade. The harvested slices were placed in distilled water in petri dish, and then their upper and lower epidermis were removed with a tweezer. The obtained transparent slices without upper and lower epidermis were selected to place in a slide center, and some distilled water were added to these slices, and then were covered with a coverslip. After above steps, the morphology structure of the transparent slice was examined by microscope. The thickness of the cuticle, the palisade tissue, the spongy tissue, the epidermal cells, and the stomata were as the observation indexes. Each index was observated 10 visual fields in the corresponding parts of slice, and its average number was counted, meanwhile, was photographed.

#### Effects of long-term alkaline stress on *P. triloba* seedling physiological indices

We separately sampled the leaf blades of *P. triloba* at 16:00 pm on day 0, 3 d, 6 d, 9 d and 12 d after the alkaline stress. The harvested leaf samples were used for the determination of the physiological activity indexes, and each index was sampled at random for three replicates. Ten physiological activity indexes were measured, including relative water content (RWC), relative electrical conductivity (REC), chlorophyll (Chl) content, soluble sugar content (SSC), soluble protein content (SPC), proline (Pro) content, malondialdehyde (MDA) content, the activity of peroxidase (POD), catalase (CAT), and superoxide dismutase (SOD). The RWC, the content of MDA, the activity of POD, and SOD were determined according to Sun and Hu [29]; the calculation of the REC was made according to Xue and Xia [30]; the chlorophyll (Chl) content was determined according to Zhu et al [31]; the SSC, SPC, Pro content, and the activity of CAT were measured according to Li [32].

#### Effects of long-term alkaline stress on *P. triloba* seedling photosynthetic parameters

The healthy and representative leaf blades of the test materials under alkaline stress and control materials on the 12th day were also used for the determination of diurnal dynamics of the photosynthetic characteristics. The photosynthetic parameters, including the net photosynthetic rate (Pn), stomatal conductance (Gs), the internal CO_2_ concentration (Ci), and transpiration rate (Tr), were measured every 2 h using a Li-6400XT portable photosynthesis system from 7:00 am to 19:00 pm. The water use efficiency (WUE) was calculated by the following formula: WUE = Pn/Tr. Each parameter was measured for nine replicates under a 3 (leaf) × 3 (plantlet) arrangement.

## Results and discussion

### *De novo* transcriptome analysis of *P. triloba* in response to short-term alkaline stress

#### Sequence analysis and assembly

**Fig. 1:**
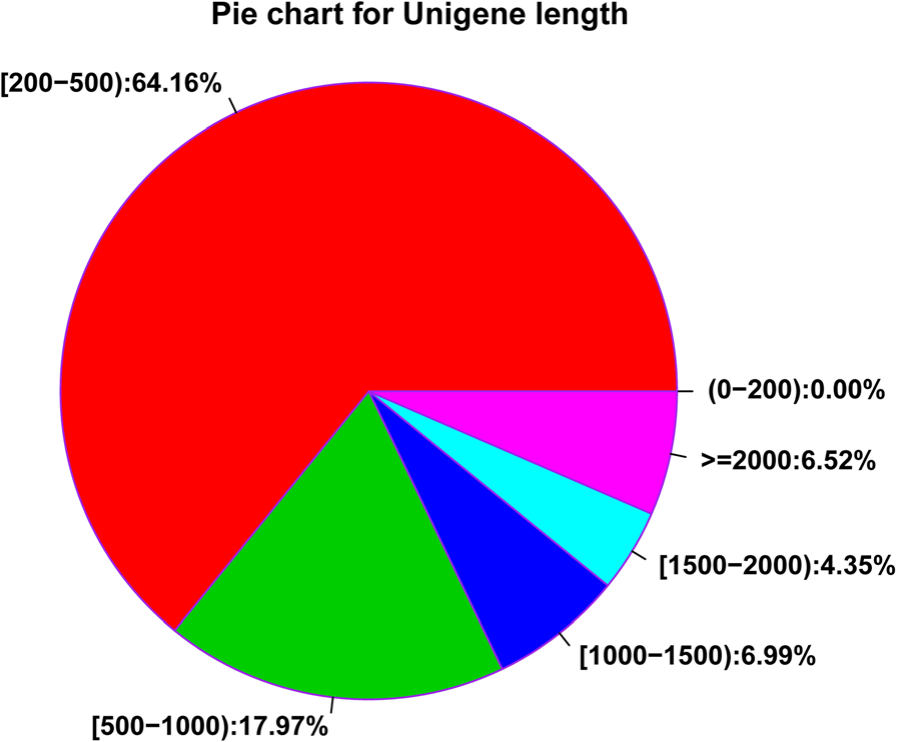
Pie chart for length distribution of the assembled unigenes

In this study, we used the Illumina HiSeq 2500 platform to obtain a total of 595,894,216 raw *P. triloba* reads during different stages of alkaline stress. These raw data were deposited in the NCBI SRA database under accession number SRP098782. After quality control trimming and filtering, there were 529,847,752 high-quality reads (~62.10 Gb clean data) remaining. The Q20, Q30, and GC content of the clean data were over 99.16, 97.69, and 45.16%, respectively, for each sample (Table 2). These results showed that our obtained clean reads were of a sufficiently high quality for subsequent analysis. Using the *de novo* assembly software Trinity and TGICL, 124,786 unigenes were obtained for all samples, with an average size of 668.65 bp and an N50 length of 1111 bp (Fig. 1). Among the assembled unigenes, 80,060 (64.16%) were 200–500 bp in length while 22,426 (17.97%) were 500–1000 bp in length (Fig. 1). To our knowledge, this is the first large-scale *P. triloba* genomic resource to be made available. The obtained transcript sequences therefore enrich the genomic resources available for *P. triloba* and can be used not only for gene discovery but also for identification of simple sequence repeat (SSR) or single nucleotide polymorphism (SNP) markers.

**Table 2:** Summary of sequences analysis

Sample	Raw reads	Clean reads	Clean bases	Q20 (%)	Q30 (%)	GC (%)
A1-0	33,244,262	30,036,644	3.51 G	99.43	98.43	46.00
A2-0	67,111,312	59,313,064	6.97 G	99.25	97.94	46.38
A1-1	75,018,602	66,678,644	7.80 G	99.28	98.03	45.41
A2-1	50,426,168	45,822,060	5.38 G	99.44	98.43	45.49
A1-3	42,772,046	38,114,654	4.45 G	99.27	97.99	46.08
A2-3	65,192,034	57,586,408	6.76 G	99.25	97.93	45.66
A1-6	46,008,224	41,153,398	4.81 G	99.27	97.98	45.46
A2-6	68,608,076	60,487,348	7.09 G	99.25	97.95	45.43
A1-12	75,873,652	67,335,634	7.90 G	99.16	97.69	45.21
A2-12	71,639,840	63,319,898	7.43 G	99.24	97.91	45.16
Total	595,894,216	529,847,752	62.10 G	–	–	–

#### Quantitative analysis of gene expression and identification of differentially expressed genes

In this study, the FPKM method was used to estimate the gene expression levels of assembled unigenes from each sample. Within the 10 samples, 20.27–32.49% of unigenes exhibited FPKM values between 0 and 1, while only 1.24–1.57% exhibited FPKM values over 60 (Additional file 1). Gene expression levels in these samples were compared by assessing the distribution diagram of the FPKMs of all genes. As shown in the Additional file 2, the density of genes among these 10 samples was highest at the log FPKM values about range within −0.30 to −0.60, and the maximum gene density was highest for sample A1-12.

**Fig. 2:**
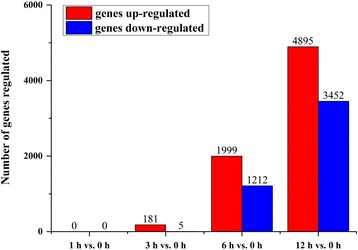
Number of DEGs at different time points after under alkaline stress compared with 0 h. Total number of up- (red bars) and down-regulated (blue bars) genes in leaves compared with the sample without stress (0 h)

Genes with an FDR threshold ≤ 0.05 and |log2 (fold change)| > 1 according to DESeq were designated as differentially expressed. In this study, among 124,786 unigenes, 8948 (7.17%) were identified as DEGs in leaves under alkaline stress. Among these, 0, 186, 3211 and 8347 DEGs were discovered through transcriptome comparison of the samples 1 h vs. 0 h, 3 h vs. 0 h, 6 h vs. 0 h, and 12 h vs. 0 h, respectively (Fig. 2). The number of DEGs found in the 12 h vs. 0 h comparison (8347) was therefore the highest. This result is inconsistent with the patterns found in *Puccinellia tenuiflor* [19] and wild soybean (*Glycine soja*) [33] roots, where the number of DEGs at 6 h was higher than that at 12 and 24 h. This difference might be attributed to the fact that the cellular response to extracellular signals in the leaves occurs later than that in the roots. It is noteworthy that no DEGs were detected until after 3 h of stress treatment in this study. This result was similar to that in wild soybean (*G. soja*) [33] roots, which also took 3 h to respond to alkaline stress at the transcription level. Of the 8948 DEGs, 181 were up-regulated in 3 h vs. 0 h; 1999 were up-regulated in 6 h vs. 0 h; and 4895 were up-regulated in 12 h vs. 0 h. In contrast, 5, 1212, and 3452 DEGs were down-regulated in 3 h vs. 0 h, 6 h vs. 0 h, and 12 h vs. 0 h, respectively. The number of up-regulated genes was greater than that of down-regulated in 3 h vs. 0 h, 6 h vs. 0 h, and 12 h vs. 0 h, which was consistent with the *Arabidopsis* transcriptome profile under various abiotic stresses [34].

#### Functional categorization of DEGs

**Fig. 3:**
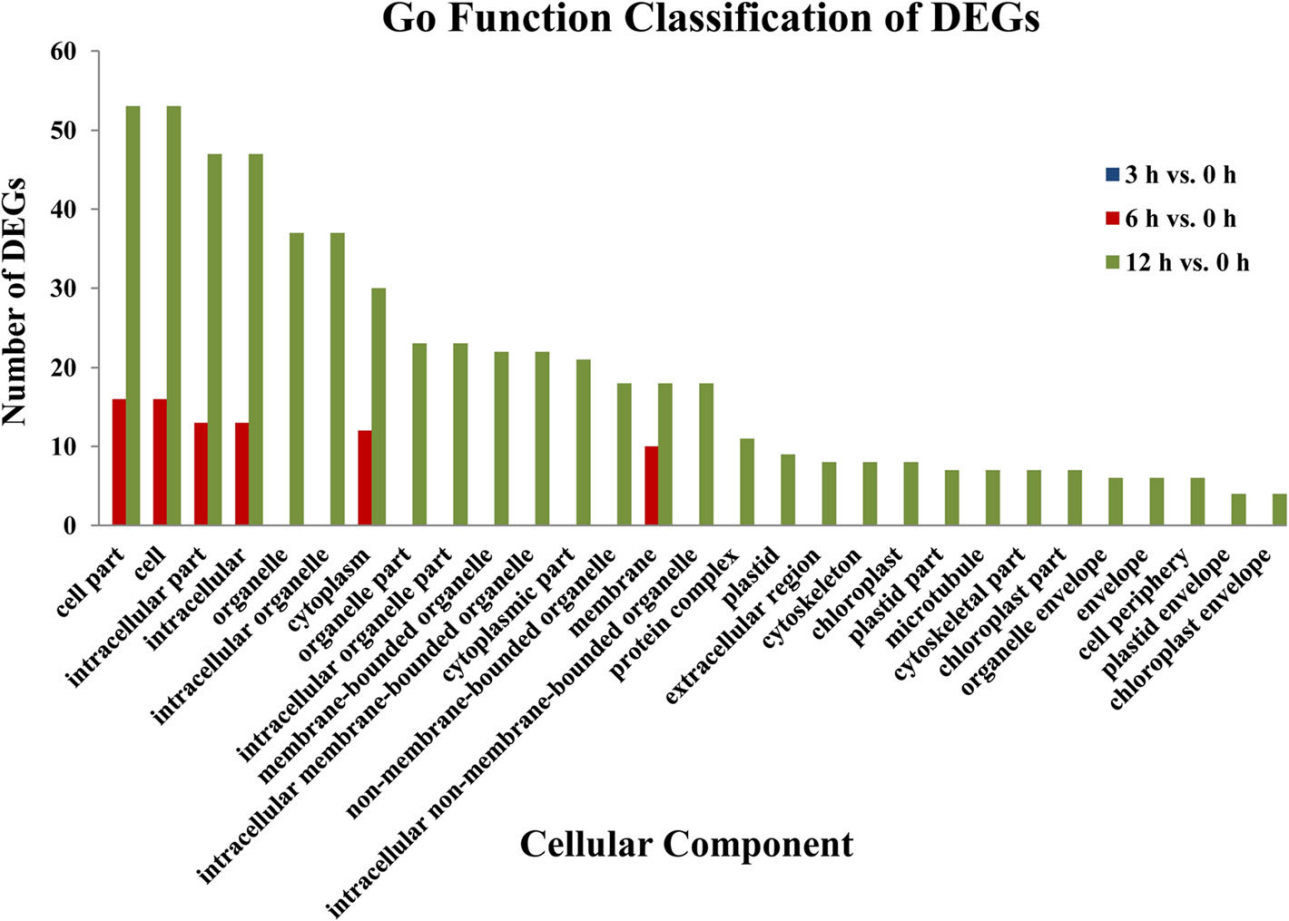
Comparative distribution of GO enrichment of terms related to cellular components

**Fig. 4:**
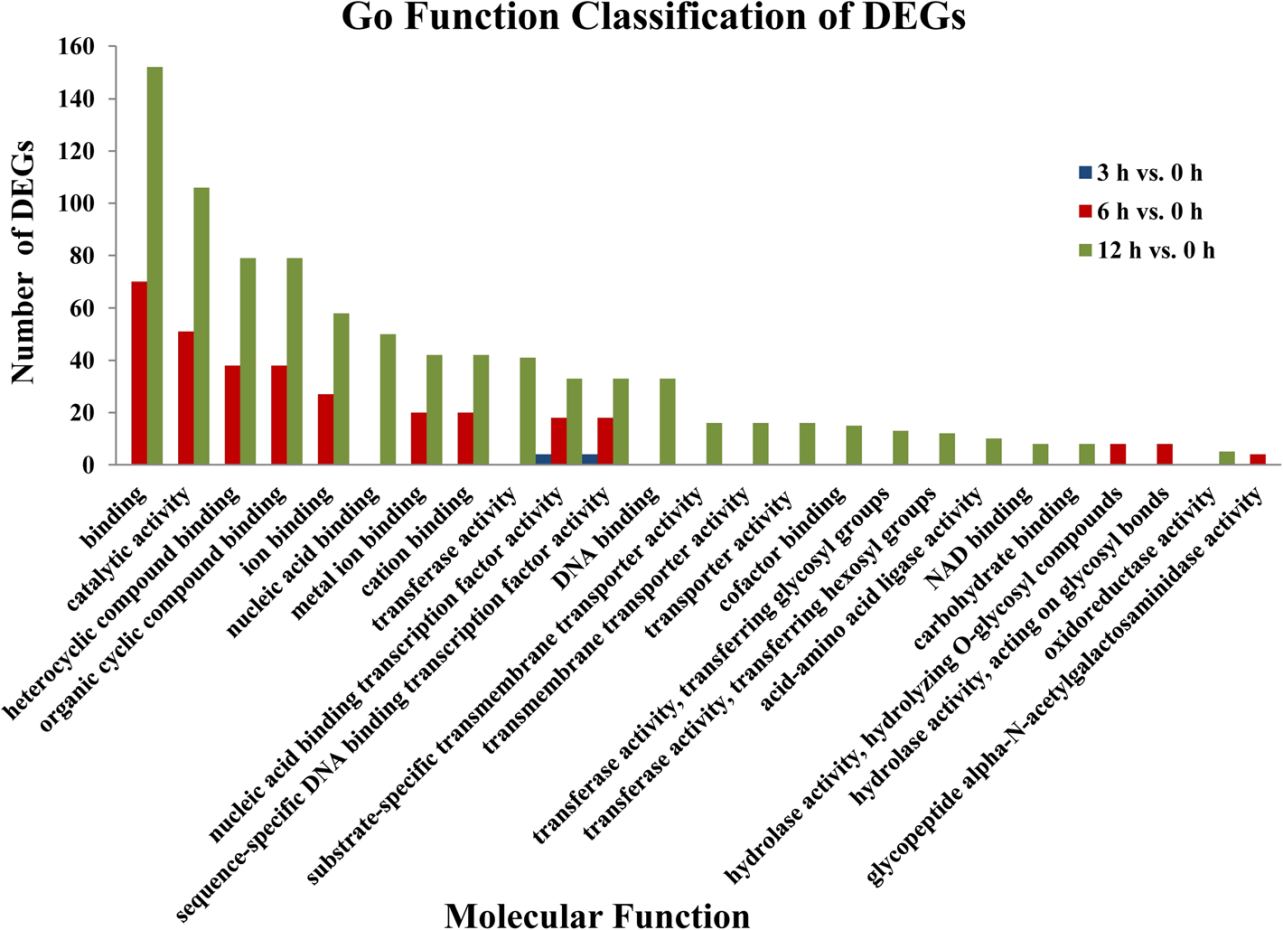
Comparative distribution of GO enrichment of terms related to molecular function

**Fig. 5:**
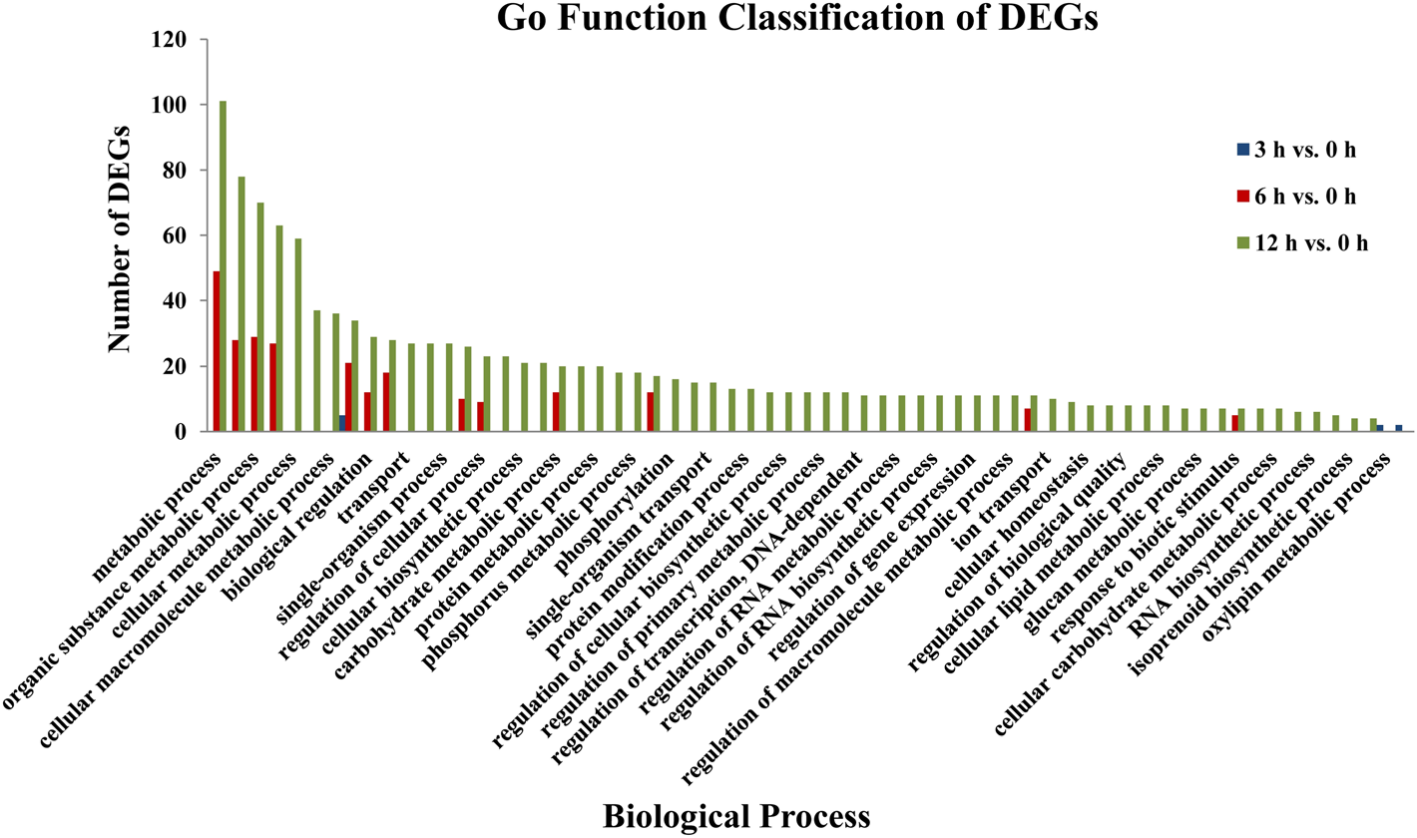
Comparative distribution of GO enrichment of terms related to biological processes

To further characterize the temporal expression changes under alkaline stress, GO enrichment analysis (*P*-value ≤ 0.01) of DEGs found in the 3 h vs. 0 h, 6 h vs. 0 h, and 12 h vs. 0 h was conducted with the whole transcriptome as the background. Analysis revealed enrichment of GO terms related to cellular components, molecular functions, and biological processes. Of the 186, 3211, and 8347 DEGs found in the 3 h vs. 0 h, 6 h vs. 0 h, and 12 h vs. 0 h comparison, respectively, 0, 24, and 72 DEGs were annotated in a GO cellular component category; 9, 146, and 307 DEGs were annotated in a GO molecular function ontology; and 11, 86, and 184 DEGs were annotated in a GO biological process category. For cellular component (Fig. 3), there was no gene annotated to the ontology term found in the 3 h vs. 0 h comparisons; whereas cell [16 (66.67%); 53 (73.61%)], cell part [16 (66.67%); 53 (73.61%)], intracellular part [13 (54.17%); 47 (65.28%)], and intracellular [13 (54.17%); 47 (65.28%)] were the most chasses for both the 6 h vs. 0 h, and 12 h vs. 0 h comparisons. In the molecular function group, as shown in Fig. 4, nucleic acid binding transcription factor activity (4, 44.44%) and sequence-specific DNA binding transcription factor activity (4, 44.44%) were the two most highly represented categories in the 3 h vs. 0 h comparisons; for the 6 h vs. 0 h and 12 h vs. 0 h comparisons, the top categories were binding [70 (47.95%); 152 (65.28%)], catalytic activity [51 (34.93%); 106 (34.53%)], heterocyclic compound binding [38 (26.03%); 79 (27.73%)], and organic cyclic compound binding [38 (26.03%); 79 (27.73%)]. Under biological process, genes from the 3 h vs. 0 h comparison were most commonly annotated as single-organism metabolic process (5, 45.45%), oxylipin metabolic process (2, 18.18%), and oxylipin biosynthetic process (2, 18.18%). Similarly, metabolic process category [49 (56.98%); 101 (54.89%)], cellular process [28 (32.56%); 78 (42.39%)], organic substance metabolic process [29 (33.72%); 70 (38.04%)], and primary metabolic process [27 (31.40%); 63 (34.24%)] were the mostly highly enriched in 6 h vs. 0 h and 12 h vs. 0 h comparisons (Fig. 5).

The results of GO enrichment analysis showed that few genes were annotated with GO ontology terms in the 3 h vs. 0 h comparison. These GO ontology terms included only nucleic acid binding transcription factor activity and sequence-specific DNA binding transcription factor activity in the molecular function group, and single-organism metabolic process, oxylipin metabolic process, and oxylipin biosynthetic process in the biological process group. These GO ontology terms showed the earliest response to alkaline stress and may generate a cascade responses following their induction at 3 h of alkaline stress treatment. Therefore, these genes may play an important role in early alkaline resistance mechanisms. With increasing duration of alkaline stress treatment, more DEGs were annotated with more GO terms, of which cell and cell part in the cellular component group, binding and catalytic activity in the molecular function group, and metabolic process category and cellular process in the biological process group were the most represented categories, suggesting that the internal tissues and organs of *P. triloba* had undergone multiple processes of anabolism to cope with and adapt to alkaline stress. It is noteworthy that 28 genes (c57635_g1_i1, c64583_g1_i1, c64900_g1_i1, c71351_g1_i1, c72539_g1_i1, c73498_g1_i4, c73839_g1_i2, c73869_g1_i2, c74641_g1_i1, c75112_g1_i1, c76093_g2_i1, c76646_g1_i1, c78345_g1_i1, c78766_g1_i1, c79591_g1_i1, c79591_g1_i2, c79591_g1_i3, c81104_g2_i1, c82384_g1_i1, c82500_g1_i1, c83082_g2_i1, c83291_g1_i3, c83618_g1_i4, c83799_g2_i1, c86513_g2_i1, c87340_g1_i1, c87510_g1_i2, c87918_g1_i2) as being involved in response to stimulus, indicating they may play a role in the alkaline stress response. KEGG annotation of these 28 genes revealed that c71351_g1_i1 is a RNA polymerase-associated protein, and c73869_g1_i2 is a HSP20 family protein. In the COG analysis of these stress-related genes, only five genes were involved in posttranslational modification, protein turnover, chaperones (c73869_g1_i2, c74641_g1_i1, c78766_g1_i1), replication, recombination and repair (c82384_g1_i1), and nucleotide transport and metabolism (c83082_g2_i1). Therefore, there appears to be a link between the putative functions and annotations of these 28 genes and the alkaline stress response in *P. triloba*.

**Fig. 6:**
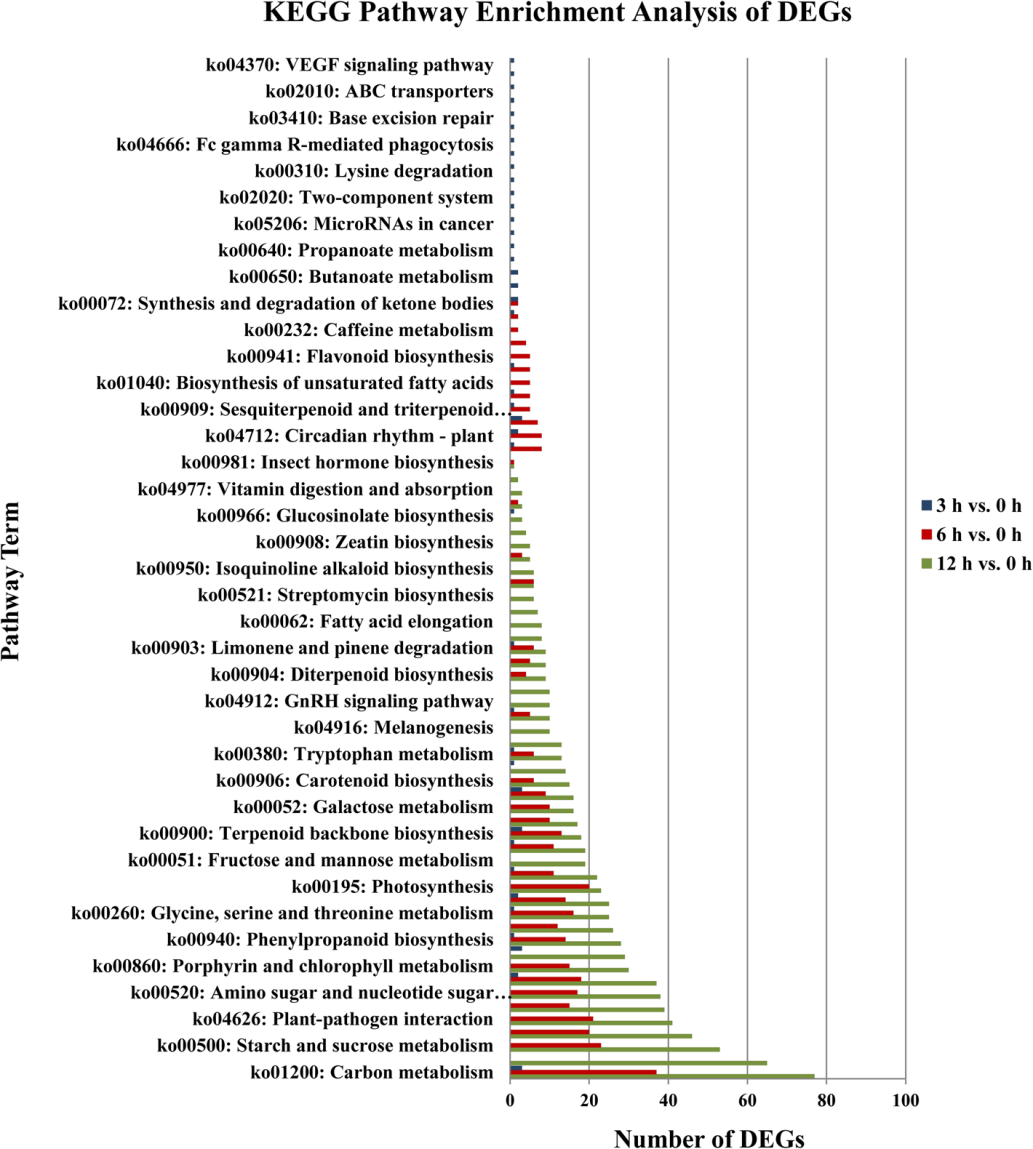
KEGG enrichment analysis of the DEGs

KEGG pathway enrichment analysis for DEGs was also conducted. There were several differences between the results of the KEGG analyses of the 3 h vs. 0 h genes, 6 h vs. 0 h genes, and 12 h vs. 0 h comparisons (Fig. 6). A total of 22, 403, and 1006 DEGs for 3 h vs. 0 h, 6 h vs. 0 h, and 12 h vs. 0 h comparison, respectively, were assigned to 42, 41, and 47 biological pathways. For 3 h vs. 0 h (Fig. 6), the largest categories were carbon metabolism (3, 13.64%), carbon fixation pathways in prokaryotes (3, 13.64%), terpenoid backbone biosynthesis (3, 13.64%), pyruvate metabolism (3, 13.64%), and alpha-linolenic acid metabolism (3, 13.64%). For 6 h vs. 0 h, as shown in Fig. 6, the top five most enriched pathway were carbon metabolism (37, 9.18%), starch and sucrose metabolism (23, 5.71%), plant-pathogen interaction (21, 5.21%), plant hormone signal transduction (20, 4.96%), and photosynthesis (20, 4.96%). In 47 assigned biological pathways of 12 h vs. 0 h comparison (Fig. 6), carbon metabolism pathway was also the most abundantly represented (77, 7.65%), followed by biosynthesis of amino acids (65, 6.46%), starch and sucrose metabolism (53, 5.27%), plant hormone signal transduction (46, 4.57%), and plant-pathogen interaction (41, 4.08%).

The results of KEGG pathways enrichment showed that metabolic pathway varied with different treatment durations. However, under alkaline stress treatment for 3 h, 6 h or 12 h, the following metabolic pathways were consistently involved: carbon metabolism; carbon fixation in photosynthetic organisms; phenylpropanoid biosynthesis; glycine, serine and threonine metabolism; methane metabolism; pyruvate metabolism; glyoxylate and dicarboxylate metabolism; terpenoid backbone biosynthesis; alpha-Linolenic acid metabolism; tryptophan metabolism; limonene and pinene degradation. Among these, the carbon metabolism pathway included the most DEGs, suggesting that *P. triloba* requires a large amount of energy in response to alkaline stress and that alkaline stress may primarily influence gene transcription by altering the expression of carbon metabolism-related genes in *P. triloba*. The pyruvate metabolism pathway involves many metabolic activities, such as glycolysis, the tricarboxylic acid cycle, and fatty acid metabolism. When the plant is exposed to a harsh external environment, the pyruvate metabolism pathway can first effectively adjust and balance the metabolic processes in vivo, then activate protective mechanism, participate in scavenging reactive oxygen species (ROS), and protect the cell membrane system, thus allowing the plant to adapt to an adverse external environment [35]. The glyoxylate and dicarboxylate metabolism pathway can balance metabolic disorders and transport energy in plants to enhance stress resistance [35]. The alpha-linolenic acid metabolism pathway produces a series of metabolites, the most important of which are DHA (docosahexaenoic acid) and EPA (eicosapentaenoic acid), as well as other polyunsaturated fatty acids. DHA and EPA increase lipid membrane fluidity and permeability and increase cells viability [36]. The tryptophan metabolism pathway produces secondary metabolites when the plant is subjected to abiotic stress, enhancing stress tolerance according to previous abiotic stress studies [37–39]. Therefore, the enrichment of DEGs in these metabolic pathways indicates that genes involved in early mechanisms of protection, defense, and stress tolerance were differentially expressed under alkaline stress treatment of 3 h. With increasing duration of alkaline stress, the number of related response genes increased, indicating that alkaline resistance in *P. triloba* is a complex train under the control of multiple genes. Some of the metabolic pathways only included DEGs after alkaline stress for a certain length of time. Under alkaline stress treatment for 3 h, one gene (c58505_g1_i1) was found to be involved in the calcium signaling pathway, and one gene (c89089_g2_i1) was involved in the ABC transporter pathway. The calcium signaling pathway is an important control pathway of physiological and biochemical reactions in vivo, and it can indirectly affect plant physiological activities (such as stomatal closure, the development of pollen tubes, root elongation, etc.), as confirmed by previous research [40]. The calcium signaling pathway was specifically identified after 3 h of alkaline stress treatment, when it may help *P. triloba* better cope with alkaline stress by changing intracellular calcium ion concentrations, leading to stomatal closure to avoid ion toxicity. ABC (ATP-binding cassette) transporters constitute the largest protein family with the greatest variety of functions, which may involve the transmembrane transport of plant secondary metabolites, protecting plants from environmental stresses [41]. The genes involved in these early specific pathways may play important roles in early alkaline resistance mechanisms and may generate a cascade of responses to subsequent alkaline stress. Under alkaline stress treatment for 6 and 12 h, more DEGs were involved in special metabolic pathways, including starch and sucrose metabolism, plant hormone signal transduction, glycolysis/gluconeogenesis, photosynthesis, carotenoid biosynthesis, and photosynthesis-antenna proteins. The above results showed that these genes that involved in metabolic pathways expressed with various patterns as the alkaline stress time increasing, and there was a rapid response of multiple molecular mechanisms allowing for the perception, transduction, and response to alkaline stress in *P. triloba*.

#### qRT-PCR validation of alkaline-related genes

**Fig. 7:**
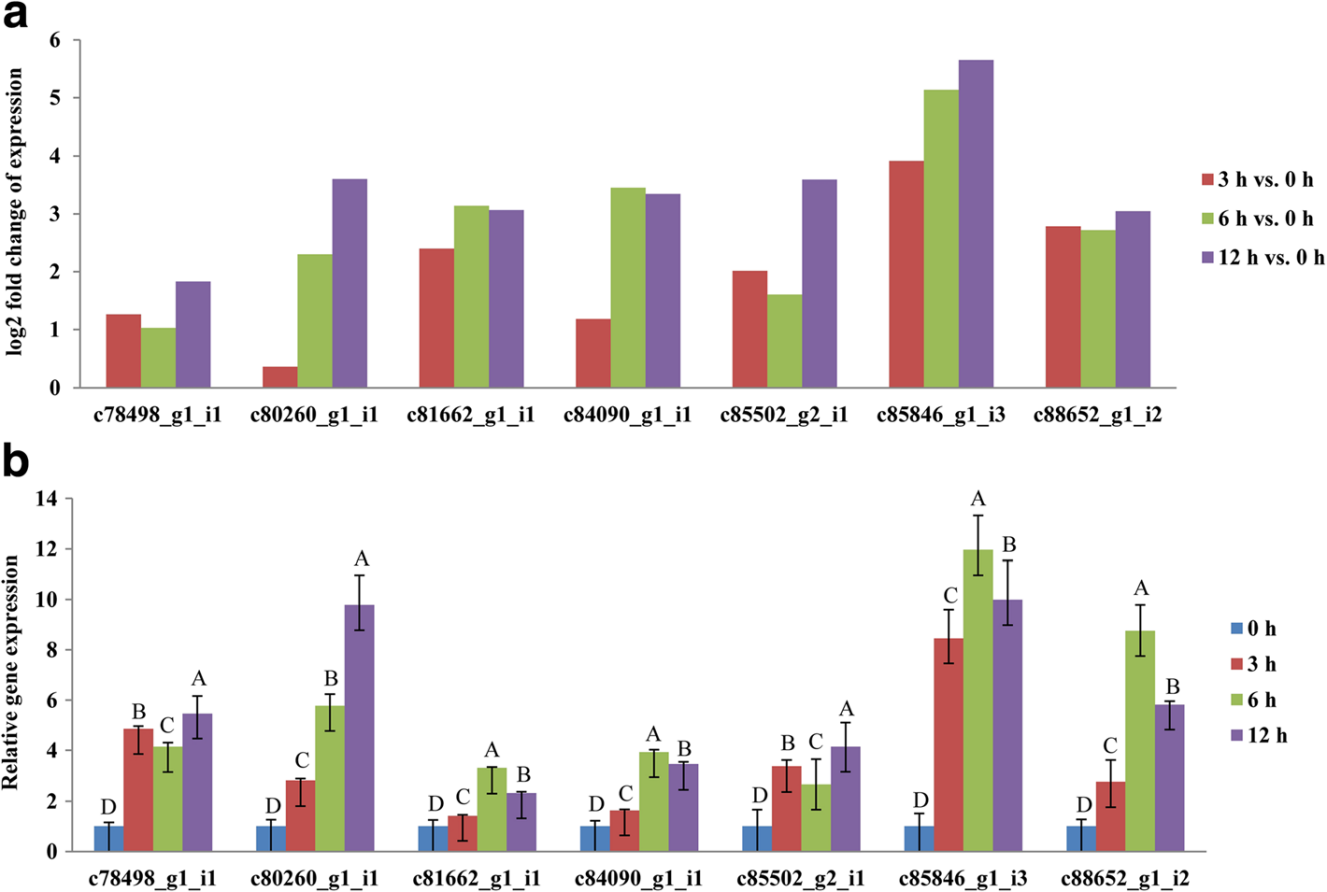
Expression patterns of seven genes selected from alkalinity-responsive DEGs of *Prunus triloba* by RNA-Seq and qRT-PCR. **a** Log2 fold change of expression was detected by RNA-Seq. **b** The relative gene expression was calculated using the 2^-△△Ct^ method by qRT-PCR. Mean values within the same gene with different letters are significantly at *P*<0.01

The reliability of the expression patterns of alkaline-responsive DEGs detected by RNA-Seq were confirmed by qRT-PCR analysis with gene-specific primers. A subset of seven genes (c78498_g1_i1, c80260_g1_i1, c81662_g1_i1, c84090_g1_i1, c85502_g2_i1, c85846_g1_i3, and c88652_g1_i2) was selected, and the actin gene was used as an internal reference gene for data normalization. RNA-Seq analysis revealed that these seven genes were all up-regulated (Fig. 7), and most of expression patterns were consistent with those determined by qRT-PCR. Some differences in the expression patterns and degree of up-regulation of the alkaline-responsive DEGs were observed between RNA-Seq and qRT-PCR, which might be due to the different algorithms and principles used by the RNA-Seq and qRT-PCR methods [42, 43]. It is thought that it is normal to find a few differences between these two methods, and our results are of a similar nature to those reported in Yates [18] and Huang (2015) [44]. They found that some genes detected by RNA-Seq also did not show similar expression patterns when compared to qRT-PCR. Moreover, all seven genes were significantly and differentially expressed at all three durations of alkaline stress (*P*<0.05), suggesting that they were involved in regulatory networks that were active during these alkaline stress stages. Taken together, similar patterns were obtained from the qRT-PCR and RNA-Seq experiments, confirming the reliability and accuracy of our sequencing methods. And for important alkaline-responsive DEGs, we will use the Northern Blot to re-validate them in the future research.

### Physiological analysis of *P. triloba* in response to long-term alkaline stress

#### Effects of long-term alkaline stress on the leaf microstructure of *P. triloba*

**Fig. 8:**
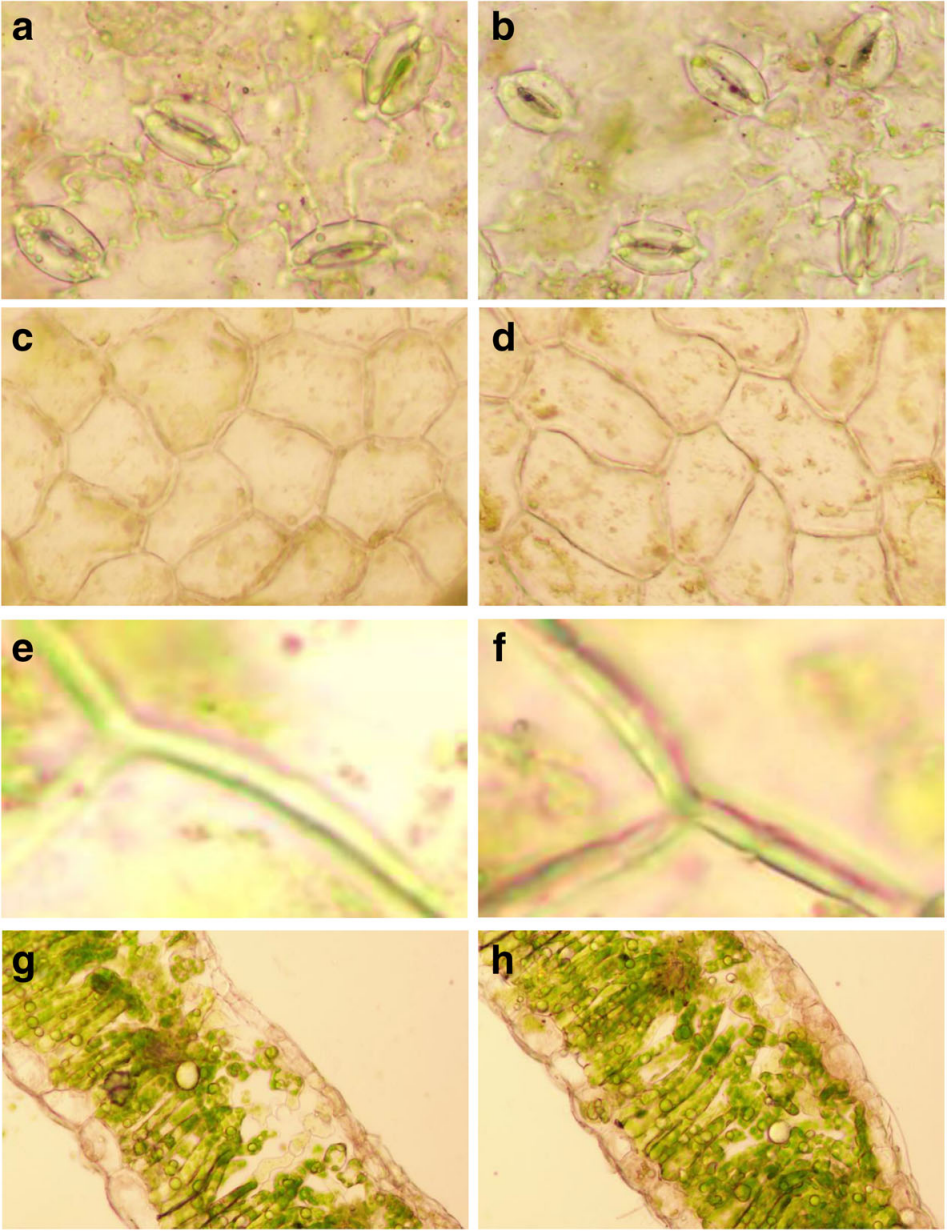
Effects of control and alkaline stress treatments on leaf microstructure of *Prunus triloba*. **a**, **c**, **e**, **g** represent the leaf stomata, upper epidermis cell, cuticle, and vertical section in the control group, repectively. **b**, **d**, **f**, **h** represent the leaf stomata, upper epidermis cell, cuticle, and vertical section in the alkaline stress group, repectively

The leaf is the main organ for plant photosynthesis and transpiration, and its structural characteristics best reflect the adaptation of plants to the environment [45]. As shown in Fig. 8, the internal microstructures of the leaves of *P. triloba* changed under alkaline stress. Compared with the control group, the main changes in the *P. triloba* leaf structure that occurred under alkaline stress can summarized as follows: 1) the stomatal density of the lower epidermis greatly increased, the stomatal volume became smaller, and the stomata almost completely closed, in contrast to the control group whose stomata were semi-closed (Fig. 8a–b); 2) the upper and lower epidermal cells became slightly longer and narrower, and their arrangement became more irregular (Fig. 8c–d); 3) the thickness of the upper and lower epidermal cuticle increased (Fig. 8e–f); and 4) The leaf thickness increased, the mesophyll structure was more compact, the leaf chamber space was significantly reduced, the palisade tissue cells became slender, the spongy tissue cells became smaller, and the thickness ratio of palisade tissue to spongy tissue increased (Fig. 8g–h).

Alkaline stress can directly affect the water metabolism of plants. It is accepted that the larger area of the epidermal cells have a better water storage, which has an important significance in enhancing the regulation of water. The epidermal cuticle can prevent excessive water transpiration inside the plant and it has an important role in the mechanical support of epidermal cells; therefore, when the plant water supply is insufficient, it keeps plant tissues from withering rapidly [46]. The highly developed palisade tissue not only helps mesophyll cells avoid scorching from strong sunlight but also effectively uses diffracted light for photosynthesis. Thicker, smaller, and more densely arranged palisade tissue cells result in a higher energy utilization efficiency in plants [46, 47]. Therefore, in this study, the leaf structural changes observed in *P. triloba*, including enlargement of the leaf epidermal cell area, thickening of the epidermal cuticle and palisade tissue, and transition to a denser arrangement of palisade tissue, reflect the plant’s responses to long-term alkaline stress.

#### Effects of long-term alkaline stress on *P. triloba* seedling physiological indices

**Fig. 9:**
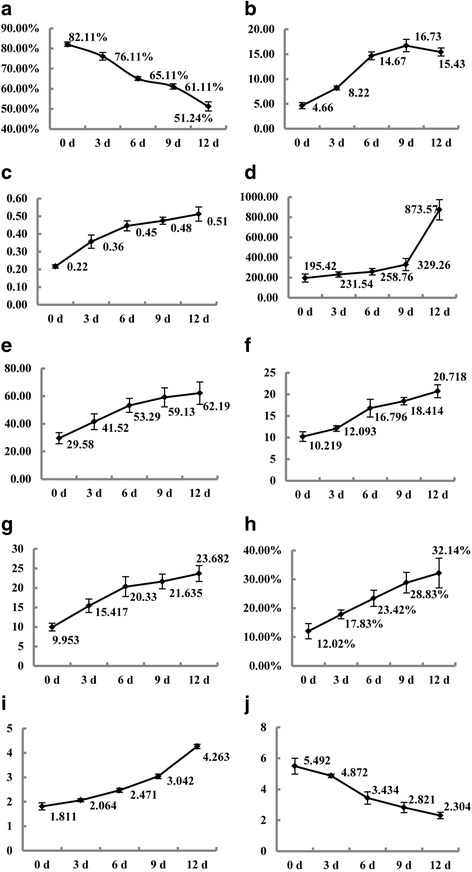
Effects of alkaline stress on seedling physiological indices of *Prunus triloba*. The x-axis represents the time (d) under alkaline stress, the y-axis are the various number of physiological indices, of which (**a**) The relative water content (RWC, %); **b** Soluble protein content (SPC, mg/g FW); **c** Soluble sugar content (SSC, mg/g); **d** Procontnet content (Pro, μg/g); **e** The activity of peroxidase (POD, μ/g FW. min); **f** The activity of catalase (CAT, μ/g FW); **g** The activity of superoxide dismutase (SOD, μ/g FW); **h** Relative electric conductivity (REC, %); **i** Malonaldehyde content (MDA, μm/g FW); **j** Total chlorophyll content (Chl, mg/g FW)

It is widely accepted that under alkaline stress, plants not only bear the same osmotic stress and ion toxicity as those under salt stress, but they also must resist damage due to high pH. In this study, *P. triloba* seedlings were continuously treated with 50 mmol/L alkaline solution for 0, 3, 6, 9, or 12 days. Leaf samples collected under alkaline stress of different durations were used to measure various physiological indices in order to explore the physiological adaptation mechanisms of early *P. triloba* seedlings under alkaline stress. The RWC is the most direct reflection of the degree of injury to seedlings under alkaline stress. The RWC of leaves declined gradually from 82.11 to 51.24% with increasing duration of alkaline stress, indicating that the cell was becoming more and more dehydrated and damage was increasing as alkaline stress continued (Fig. 9a).

Osmotic stress in plants is due to loss of water or difficulty in absorbing water, so the osmotic regulation of plant cells is a basic adaptation that improves stress tolerance [48]. Osmotic protective substances produced in the cytoplasm are induced by saline-alkaline stress and consist mainly of low-molecular-weight cell-compatible organic substances (i.e. soluble sugars, soluble proteins, and proline); these maintain the proper osmotic potential of the cell, protecting it from dehydration and stabilizing the structure and function of biological macromolecules in cells [49]. In this study, the SSC, SPC, and Pro content increased with increasing stress duration (Fig. 9b–d).

After osmotic stress and ion toxicity, further damage in plants is caused by saline-alkaline stress due to excessive accumulation of ROS, resulting in lipid peroxidation or even cell death [50]. Our results suggested that 50 mmol/L alkaline solution induced the accumulation of ROS and the oxidative damage of membrane lipids in *P. triloba* seedlings and that the extent of the damage increased with an increasing duration of alkaline stress. The antioxidant systems of plants evolved to play an important role in defending against damage caused by ROS [51]. The protective enzymes in the enzyme-promoting defense system, including SOD, POD, and CAT, are particularly important. As shown in Fig. 9e–g, the activities of SOD, POD, and CAT in the leaves of *P. triloba* seedlings all increased with an increasing duration of alkaline stress. The biggest increase in activity was exhibited by POD, followed by SOD and then CAT, suggesting that POD played the primary role in resisting oxidative damage induced by alkaline stress.

The protoplasmic membrane is sensitive to the stress reaction, and an increase in cell membrane permeability is closely related to an adverse external environment [52]. The REC is an indicator of cell membrane permeability, and it can reflect the degree of cell membrane damage [53]. Moreover, MDA is produced during cell membrane lipid peroxidation. The MDA content can therefore reflect the degree of oxidative damage in an organism [54]. Our results showed that as the duration of alkaline stress increased, leaf REC and MDA content both increased gradually (Fig. 9h–i), indicating that plant damage was becoming more severe. MDA not only has a cytotoxic effect by damaging cell membrane structure, but it can also degrade chlorophyll, thereby reducing plant photosynthesis [55]. The Chl content in this study decreased gradually, from 5.492 to 2.304 mg/g, as the duration of alkaline stress increased (Fig. 9j). This may be due to the increasing MDA content, which may have inhibited the synthesis of chlorophyll.

#### Effects of long-term alkaline stress on *P. triloba* seedling photosynthetic parameters

**Fig. 10:**
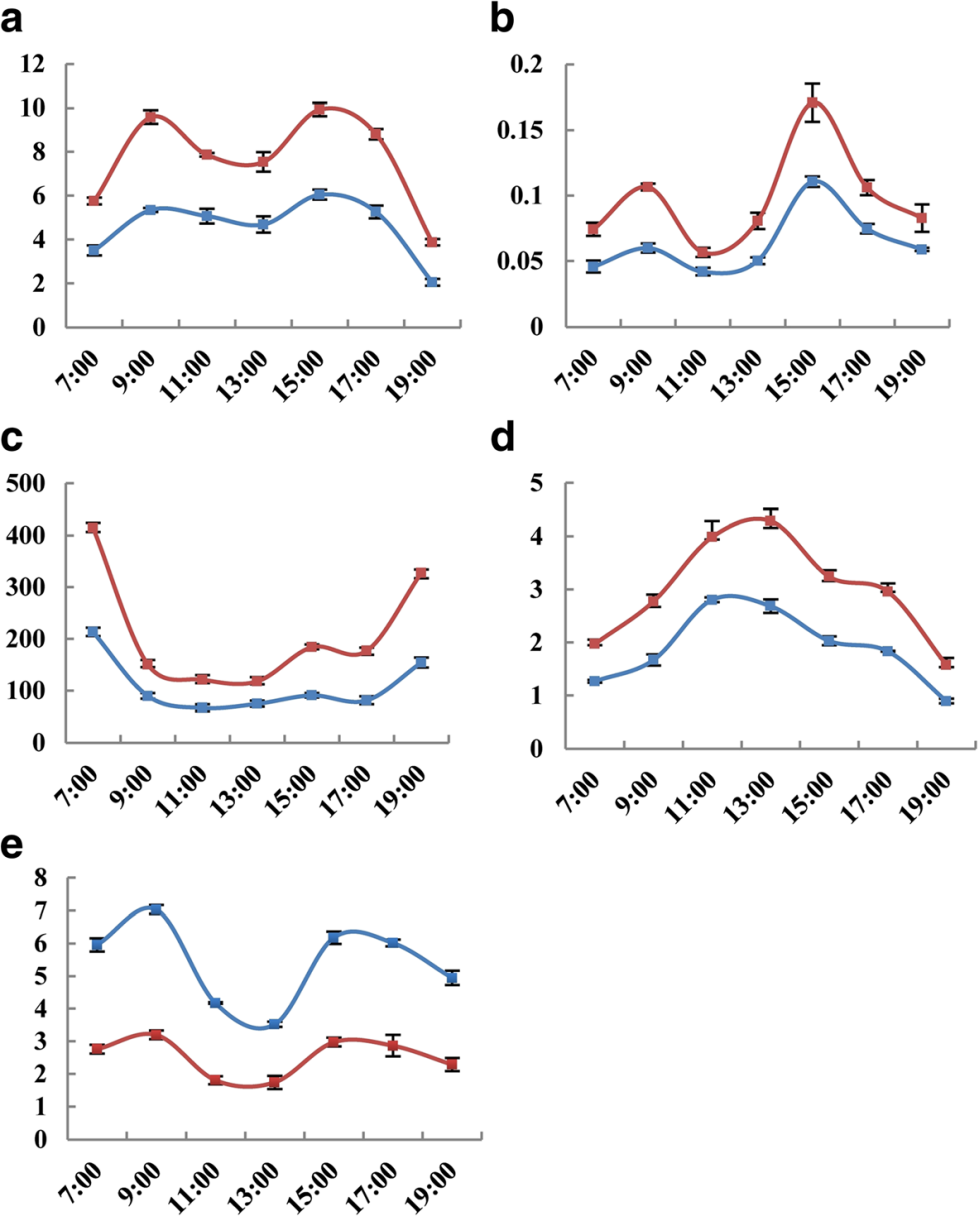
Effects of alkaline stress on seedling photosynthetic characteristics of *Prunus triloba*. The x-axis represents the measured time (h), the y-axis are the photosynthetic parameters content of the control and treatment materials, of which (**a**) Net photosynthetic rate (Pn, μmol· m^−2^· s^−1^); **b** Stomatal conductance (Gs, mmol· m^−2^· s^−1^); **c** Intercellular CO_2_ concentration (Ci, μL· L^−1^); **d** Transpiration rate (Tr, mmol· m^−2^· s^−1^); **e** Water use efficiency (WUE, Pn/Tr). The red smooth curve in the picture represents the control material, whereas the blue smooth curve represents the treatment material

The chlorophyll content is an important parameter that reflects the photosynthetic function of leaves. As mentioned above, the Chl content declined gradually with increasing stress duration. In addition, other photosynthetic parameters declined after 12 days of alkaline stress, including Pn, Gs, Ci, and Tr. Among these parameters, Gs is closely related to the photosynthetic rate of plants. In most cases, a decline in Gs leads to a decrease in CO_2_ supply, causing a reduction in the photosynthetic rate [56]. Our results showed that the Pn rate and Gs of leaves of experimental and control seedlings presented similar bimodal changes as the duration of stress increased (Fig. 10a–b), demonstrating that the change in the Pn rate was largely determined by stomatal elements.

As shown in Fig. 10d, the leaf Tr of the alkaline-stressed and control seedlings first increased and then decreased, peaking at 11:00 and 13:00, respectively, and exhibiting similar but also different trends compared with that of Pn rate and Gs. The change in Ci under alkaline stress was similar to that in the control group, which is inconsistent with the corresponding trend of the Pn rate (Fig. 10c). At some time points, the Ci and Pn rate showed similar trends (both increasing or both decreasing), while at other time points, the Ci and Pn rate exhibited opposite trends, suggesting that the decrease in the Pn rate was not only due to a stomatal limitation but was also restricted by non-stomatal factors. WUE is a comprehensive physiological and ecological index that reflects crop energy conversion efficiency in crop production and assesses crop growth suitability. The WUE at the single leaf level is often expressed as the ratio of the Pn rate to the Tr [57]. The trend in the WUE of the leaves of *P. triloba* seedlings was consistent with that of the Pn rate both in the control and alkaline stress treatments (Fig. 10e). However, the alkaline stress treatment increased the WUE in *P. triloba* leaves, a phenomenon that needed to be explained by further research.

## Conclusion

In this study, we conducted a comprehensive investigation of alkaline acclimation mechanisms in *P. triloba.* First, *de novo* transcriptome analysis of *P. triloba* was conducted in response to short-term alkaline stress using the Illumina sequencing platform. We identified a large number of DEGs that may be used for molecular breeding of *P. triloba* in the future. GO annotation analysis revealed 28 DEGs that may play an important role in the early alkaline stress response, and KEGG pathway analysis revealed a variety of pathways that may be important at different time points. In addition, the expression patterns of seven alkaline-related genes were validated with qRT-PCR, confirming the reliability of the RNA-Seq results. Second, a physiological analysis of *P. triloba* in response to long-term alkaline stress was also conducted. Changes in the internal microstructure of the leaves, physiological indices, and photosynthetic parameters of *P. triloba* under alkaline stress were observed, allowing *P. triloba* to adapt to long-term alkaline stress. Our study provides information on the short-term molecular mechanisms and long-term physiological mechanisms of alkaline tolerance in *P. triloba* that can be used to study this plant as well as other fruit trees in the future.

## Additional files

Additional file 1: Gene number of each sample in different interval of expression level. (DOC 37 kb)

Additional file 2: The comparison distribution diagram of FPKM of all genes in the 10 samples. (JPG 91 kb)

## Abbreviations

CAT: Catalase
Chl: Chlorophyll content
Ci: Internal CO_2_ concentration
DEGs: Differentially expressed genes
FDR: False discovery rate
GO: Gene ontology
Gs: Stomatal conductance
KEGG: Kyoto encyclopedia of genes and genomes
MDA: Malondialdehyde content
Pn: Net photosynthetic rate
POD: Peroxidase
Pro: Proline content
qRT-PCR: Quantitative real-time reverse-transcriptase PCR
REC: Relative electrical conductivity
RWC: Relative water content
SNP: Single nucleotide polymorphism
SOD: Superoxide dismutase
SPC: Soluble protein content
SSC: Soluble sugar content
SSR: Simple sequence repeat
Tr: Transpiration rate
WUE: Water use efficiency
