# Supplementary material for: Analysis of differentially expressed genes and adaptive mechanisms of Prunus triloba Lindl. under alkaline stress
Source: Hereditas. 2017 May 4;154:10. doi: 10.1186/s41065-017-0031-7 (PMC5418693; doi:10.1186/s41065-017-0031-7)
Supplement: Supplementary file 1 — Gene number of each sample in different interval of expression level. (DOC 37 kb) [file 41065_2017_31_MOESM1_ESM.doc]

**Additional file 1:** Gene number of each sample in different interval of expression level.

| **FPKM interval**  **Samples** | **0-1** | **1-3** | **3-5** | **15-60** | **>60** |
| --- | --- | --- | --- | --- | --- |
| A1-0 | 25,319 (20.29%) | 12,583 (10.08%) | 10,641 (8.53%) | 4,719 (3.78%) | 1,548 (1.24%) |
| A2-0 | 30,230 (24.23%) | 7,466 (5.98%) | 8,642 (6.93%) | 3,942 (3.16%) | 1,549 (1.24%) |
| A1-1 | 39,220 (31.43%) | 10,183 (8.16%) | 9,765 (7.83%) | 4,199 (3.36%) | 1,619 (1.30%) |
| A2-1 | 33,491 (26.84%) | 10,625 (8.51%) | 10,424 (8.35%) | 4,770 (3.82%) | 1,731 (1.39%) |
| A1-3 | 25,296 (20.27%) | 9,313 (7.46%) | 8,934 (7.16%) | 4,539 (3.64%) | 1,788 (1.43%) |
| A2-3 | 34,295 (27.48%) | 9,042 (7.25%) | 8,138 (6.52%) | 4,096 (3.28%) | 1,748 (1.40%) |
| A1-6 | 29,853 (23.92%) | 9,325 (7.47%) | 8,918 (7.15%) | 4,395 (3.52%) | 1,885 (1.51%) |
| A2-6 | 30,678 (24.58%) | 7,752 (6.21%) | 7,969 (6.39%) | 3,972 (3.18%) | 1,963 (1.57%) |
| A1-12 | 40,547 (32.49%) | 9,291 (7.45%) | 8,819 (7.07%) | 4,143 (3.32%) | 1,933 (1.55%) |
| A2-12 | 36,729 (29.43%) | 8,929 (7.16%) | 8,572 (6.87%) | 4,127 (3.31%) | 1,949 (1.56%) |
