# Supplementary figures and images for: Analysis of differentially expressed genes and adaptive mechanisms of Prunus triloba Lindl. under alkaline stress
Source: Hereditas. 2017 May 4;154:10. doi: 10.1186/s41065-017-0031-7 (PMC5418693; doi:10.1186/s41065-017-0031-7)

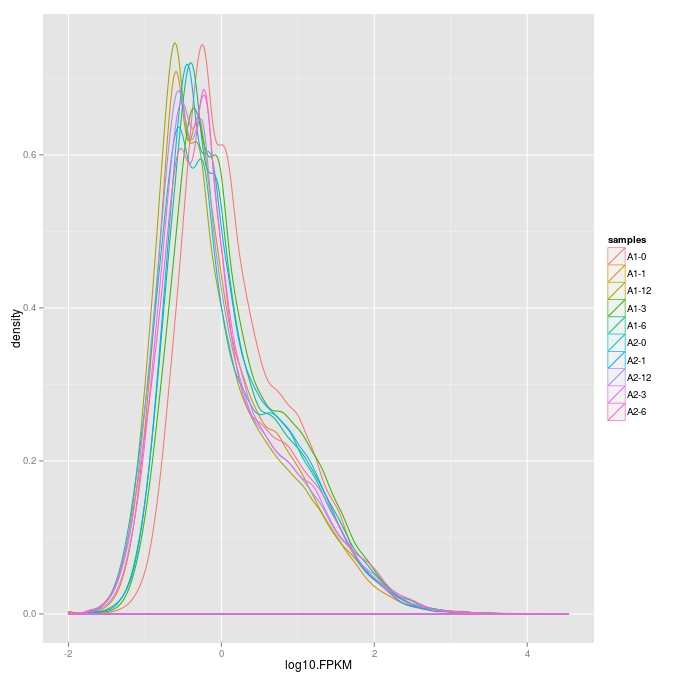

Supplement: Supplementary file 2 — The comparison distribution diagram of FPKM of all genes in the 10 samples. (JPG 91 kb) [file 41065_2017_31_MOESM2_ESM.jpg]
